# Supplementary material for: Modelling Creativity: Identifying Key Components through a Corpus-Based Approach
Source: PLoS One. 2016 Oct 5;11(10):e0162959. doi: 10.1371/journal.pone.0162959 (PMC5051932; doi:10.1371/journal.pone.0162959)
Supplement: S1 Appendix — These 30 papers were used as the creativity corpus for this work. (PDF) [file pone.0162959.s001.pdf]

Non-Creativity Corpus

- C. Ames and J. Archer. Achievement goals in the classroom: Students' learning strategies and motivation processes. Journal of Educational Psychology, 80(3):260-267, 1988.
- J. Anderson and D. Gerbing. Structural equation modeling in practice: A review and recommended two-step approach. Psychological Bulletin, 103(3):411-423, 1988.
- J. Arnett. Emerging adulthood: A theory of development from the late teens through the twenties. American Psychologist, 55(5):469-480, 2000.
- M. Arulampalam, S. Maskell, N. Gordon, and T. Clapp. A tutorial on particle filters for online nonlinear/non-gaussian bayesian tracking. IEEE Transactions on Signal Processing, 50(2):174-188, 2002.
- A. Baddeley. Exploring the central executive. Quarterly Journal of Experimental Psychology Section A: Human Experimental Psychology, 49(1):5-28, 1996.
- T. Baker, M. Piper, D. McCarthy, M. Majeskie, and M. Fiore. Addiction motivation reformulated: An affective processing model of negative reinforcement. Psychological Review, 111(1):33-51, 2004.
- P. Barnett and I. Gotlib. Psychosocial functioning and depression: Distinguishing among antecedents, concomitants, and consequences. Psychological Bulletin, 104(1):97-126, 1988.
- J. Baron. Nonconsequentialist decisions. Behavioral and Brain Sciences, 17(1):1-42, 1994.
- F. Beach. The snark was a boojum. American Psychologist, 5(4):115-124, 1950.
- M. Belkin, P. Niyogi, and V. Sindhwani. Manifold regularization: A geometric framework for learning from labeled and unlabeled examples. Journal of Machine Learning Research, 7:2399-2434, 2006.
- G. Bonanno. Loss, trauma, and human resilience: Have we underestimated the human capacity to thrive after extremely aversive events? American Psychologist, 59(1):20-28, 2004.
- T. Chan and L. Vese. Active contours without edges. IEEE Transactions on Image Processing, 10(2):266-277, 2001.
- H. Cheng and J. Schweitzer. Cultural values reflected in Chinese and U.S. television commercials. Journal of Advertising Research, 36(3):27-45, 1996.

- C. Coello Coello. Evolutionary multi-objective optimization: A historical view of the field. IEEE Computational Intelligence Magazine, 1(1):28-36, 2006.
- D. Comaniciu and P. Meer. Mean shift: A robust approach toward feature space analysis. IEEE Transactions on Pattern Analysis and Machine Intelligence, 24(5):603-619, 2002.
- L. Cronbach and L. Furby. How we should measure 'change': Or should we? Psychological Bulletin, 74(1):68-80, 1970.
- M. Davis. Measuring individual differences in empathy: Evidence for a multidimensional approach. Journal of Personality and Social Psychology, 44(1):113-126, 1983.
- J. Demšar. Statistical comparisons of classifiers over multiple data sets. Journal of Machine Learning Research, 7:1-30, 2006.
- M. Dorigo, M. Birattari, and T. Stützle. Ant colony optimization artificial ants as a computational intelligence technique. IEEE Computational Intelligence Magazine, 1(4):28-39, 2006.
- E. Fischer and J. Turner. Orientations to seeking professional help: Development and research utility of an attitude scale. Journal of Consulting and Clinical Psychology, 35(1 PART 1):79-90, 1970.
- J. Gibson. Observations on active touch. Psychological Review, 69(6):477-491, 1962.
- A. Gopnik and J. Astington. Children's understanding of representational change and its relation to the understanding of false belief and the appearance-reality distinction. Child development, 59(1):26-37, 1988.
- S. Gosling, S. Vazire, S. Srivastava, and O. John. Should we trust web-based studies? A comparative analysis of six preconceptions about internet questionnaires. American Psychologist, 59(2):93-104, 2004.
- J. Gray. The psychophysiological basis of introversion-extraversion. Behaviour Research and Therapy, 8(3):249-266, 1970.
- B. Grosz and S. Kraus. Collaborative plans for complex group action. Artificial Intelligence, 86(2):269- 357, 1996.
- P. Groves and R. Thompson. Habituation: A dual-process theory. Psychological Review, 77(5):419-450, 1970.
- M. Hall, J. Anderson, S. Amarasinghe, B. Murphy, S.-W. Liao, E. Bugnion, and M. Lam. Maximizing multiprocessor performance with the SUIF compiler. Computer, 29(12):84-89, 1996.

- F. Happé. The role of age and verbal ability in the theory of mind task performance of subjects with autism. Child development, 66(3):843-855, 1995.

- C. Harland. Supply chain management: Relationships, chains and networks. British Journal of Management, 7(SPEC. ISS.):S63-S80, 1996.

- S. Hayes, K. Strosahl, K. Wilson, R. Bissett, J. Pistorello, D. Toarmino, M. Polusny, T. Dykstra, S. Batten, J. Bergan, S. Stewart, M. Zvolensky, G. Eifert, F. Bond, J. Forsyth, M. Karekla, and S. McCurry. Measuring experiential avoidance: A preliminary test of a working model. Psychological Record, 54(4):553-578, 2004.

- J. Hirsch and K. Lücke. Overview no. 76. mechanism of deformation and development of rolling textures in polycrystalline f.c.c. metals-i. description of rolling texture development in homogeneous cuzn alloys. Acta Metallurgica, 36(11):2863-2882, 1988.

- P. Killeen. Mathematical principles of reinforcement. Behavioral and Brain Sciences, 17(1):105-172, 1994.

- P. Kirschner, J. Sweller, and R. Clark. Why minimal guidance during instruction does not work: An analysis of the failure of constructivist, discovery, problem-based, experiential, and inquiry-based teaching. Educational Psychologist, 41(2):75-86, 2006.

- S. Liao. Notes on the homotopy analysis method: Some definitions and theorems. Communications in Nonlinear Science and Numerical Simulation, 14(4):983-997, 2009.

- C. Lord, L. Ross, and M. Lepper. Biased assimilation and attitude polarization: The effects of prior theories on subsequently considered evidence. Journal of Personality and Social Psychology, 37(11):2098- 2109, 1979.

- S. Luthar, D. Cicchetti, and B. Becker. The construct of resilience: A critical evaluation and guidelines for future work. Child Development, 71(3):543-562, 2000.

- G. Mandler. Recognizing: The judgment of previous occurrence. Psychological Review, 87(3):252-271, 1980.

- G. Miller and J. Selfridge. Verbal context and the recall of meaningful material. The American journal of psychology, 63(2):176-185, 1950.

- S. Miller. Monitoring and blunting: Validation of a questionnaire to assess styles of information seeking under threat. Journal of Personality and Social Psychology, 52(2):345-353, 1987.

- G. Navarro and V. Mäkinen. Compressed full-text indexes. ACM Computing Surveys, 39(1), 2007.

- M. Nissen and P. Bullemer. Attentional requirements of learning: Evidence from performance measures. Cognitive Psychology, 19(1):1-32, 1987.
- J. Payne, J. Bettman, and E. Johnson. Adaptive strategy selection in decision making. Journal of Experimental Psychology: Learning, Memory, and Cognition, 14(3):534-552, 1988.
- J. Prochaska, C. DiClemente, and J. Norcross. In search of how people change: Applications to addictive behaviors. American Psychologist, 47(9):1102-1114, 1992.
- T. Richardson, M. Shokrollahi, and R. Urbanke. Design of capacity-approaching irregular low-density parity-check codes. IEEE Transactions on Information Theory, 47(2):619-637, 2001.
- W. Rozeboom. The fallacy of the null-hypothesis significance test. Psychological Bulletin, 57(5):416-428, 1960.
- C. Rusbult. A longitudinal test of the investment model: The development (and deterioration) of satisfaction and commitment in heterosexual involvements. Journal of Personality and Social Psychology, 45(1):101-117, 1983.
- T. Ryan. Significance tests for multiple comparison of proportion, variance, and other statistics. Psychological Bulletin, 57(4):318-328, 1960.
- W. Schultz. Behavioral theories and the neurophysiology of reward. Annual Review of Psychology, 57:87-115, 2006.
- E. Sirin, B. Parsia, B. Grau, A. Kalyanpur, and Y. Katz. Pellet: A practical OWL-DL reasoner. Web Semantics, 5(2):51-53, 2007.
- T. Srull and R. Wyer. The role of category accessibility in the interpretation of information about persons: Some determinants and implications. Journal of Personality and Social Psychology, 37(10):1660- 1672, 1979.
- J. Steiger. Tests for comparing elements of a correlation matrix. Psychological Bulletin, 87(2):245-251, 1980.
- L. Steinberg, S. Lamborn, S. Dornbusch, and N. Darling. Impact of parenting practices on adolescent achievement: authoritative parenting, school involvement, and encouragement to succeed. Child development, 63(5):1266-1281, 1992.
- D. Tao, X. Li, X. Wu, W. Hu, and S. Maybank. Supervised tensor learning. Knowledge and Information Systems, 13(1):1-42, 2007.
- A. Tellegen, D. Lykken, T. Bouchard Jr., K. Wilcox, N. Segal, and S. Rich. Personality similarity in twins reared apart and together. Journal of Personality and Social Psychology, 54(6):1031-1039, 1988.

- L. Thomas and D. Ganster. Impact of family-supportive work variables on work-family conflict and strain: A control perspective. Journal of Applied Psychology, 80(1):6-15, 1995.
- I. Thompson. Coupled reaction channels calculations in nuclear physics. Computer Physics Reports, 7(4):167-212, 1988.
- E. Tulving. Subjective organization in free recall of "unrelated" words. Psychological Review, 69(4):344- 354, 1962.
- U. Von Luxburg. A tutorial on spectral clustering. Statistics and Computing, 17(4):395-416, 2007.
- J. Williams, A. Mathews, and C. MacLeod. The emotional stroop task and psychopathology. Psychological Bulletin, 122(1):3-24, 1996.
- J. Wright, A. Yang, A. Ganesh, S. Sastry, and Y. Ma. Robust face recognition via sparse representation. IEEE Transactions on Pattern Analysis and Machine Intelligence, 31(2):210-227, 2009.
