# Supplementary material for: Modelling Creativity: Identifying Key Components through a Corpus-Based Approach
Source: PLoS One. 2016 Oct 5;11(10):e0162959. doi: 10.1371/journal.pone.0162959 (PMC5051932; doi:10.1371/journal.pone.0162959)
Supplement: S2 Appendix — These 60 papers were used as the non-creativity corpus for this work. (PDF) [file pone.0162959.s002.pdf]

Creativity Corpus

- T. M. Amabile. The social psychology of creativity: A componential conceptualization. Journal of Personality and Social Psychology, 45(2):357-376, 1983.
- M. A. Boden. Precis of The Creative Mind: Myths and mechanisms. Behavioural and Brain Sciences, 17(3):519-570, 1994.
- D. T. Campbell. Blind variation and selective retentions in creative thought as in other knowledge processes. Psychological Review, 67(7):380-400, 1960.
- S. Colton, A. Pease, and G. Ritchie. The effect of input knowledge on creativity. In Proceedings of Workshop Program of ICCBR-Creative Systems: Approaches to Creativity in AI and Cognitive Science, 2001.
- M. Csikszentmihalyi. Motivation and creativity: Toward a synthesis of structural and energistic approaches to cognition. New Ideas in Psychology, 6(2):159-176, 1988.
- M. Dellas and E. L. Gaier. Identification of creativity: The individual. Psychological Bulletin, 73(1):55- 73, 1970.
- A. Dietrich. The cognitive neuroscience of creativity. Psychonomic Bulletin & Review, 11(6):1011-1026, 2004.
- G. Domino. Identification of potentially creative persons from the adjective check list. Journal of Consulting and Clinical Psychology, 35(1):48-51, 1970.
- W. Duch. Intuition, insight, imagination and creativity. IEEE Computational Intelligence Magazine, 2(3):40-52, 2007.
- C. S. Findlay and C. J. Lumsden. The creative mind: Toward an evolutionary theory of discovery and innovation. Journal of Social and Biological Systems, 11(1):3-55, 1988.
- C. M. Ford. A theory of individual creative action in multiple social domains. The Academy of Management Review, 21(4):1112-1142, 1996.
- J. Gero. Creativity, emergence and evolution in design. Knowledge-Based Systems, 9(7):435-448, 1996.
- H. G. Gough. A creative personality scale for the adjective checklist. Journal of Personality and Social Psychology, 37(8):1398-1405, 1979.
- J. P. Guilford. Creativity. American Psychologist, 5:444-454, 1950.
- Z. Ivcevic. Creativity map: Toward the next generation of theories of creativity. Psychology of Aesthetics, Creativity, and the Arts, 3(1):17-21, 2009.

- K. H. Kim. Can we trust creativity tests? A review of the Torrance tests of creative thinking (TTCT). Creativity Research Journal, 18(1):3-14, 2006.
- L. A. King, L. McKee Walker, and S. J. Broyles. Creativity and the five-factor model. Journal of Research in Personality, 30(2):189-203, 1996.
- R. R. McCrae. Creativity, divergent thinking, and openness to experience. Journal of Personality and Social Psychology, 52(6):1258-1265, 1987.
- S. A. Mednick. The associative basis of the creative process. Psychological Review, 69(3):220-232, 1962.
- M. D. Mumford and S. B. Gustafson. Creativity syndrome: Integration, application, and innovation. Psychological Bulletin, 103(1):27-43, 1988.
- M. T. Pearce, D. Meredith, and G. A. Wiggins. Motivations and methodologies for automation of the compositional process. Musicae Scientae, 6(2):119-147, 2002.
- J. A. Plucker, R. A. Beghetto, and G. T. Dow. Why isn't creativity more important to educational psychologists? Potentials, pitfalls, and future directions in creativity research. Educational Psychologist, 39(2):83-96, 2004.
- R. Richards, D. K. Kinney, M. Benet, and A. P. C. Merzel. Assessing everyday creativity: Characteristics of the lifetime creativity scales and validation with three large samples. Journal of Personality and Social Psychology, 54(3):476-485, 1988.
- G. Ritchie. The transformational creativity hypothesis. New Generation Computing, 24(3):241-266, 2006.
- G. Ritchie. Some empirical criteria for attributing creativity to a computer program. Minds and Machines, 17:67-99, 2007.
- D. L. Rubenson and M. A. Runco. The psychoeconomic approach to creativity. New Ideas in Psychology, 10(2):131-147, 1992.
- M. A. Runco and I. Chand. Cognition and creativity. Educational Psychology Review, 7(3):243-267, 1995.
- D. K. Simonton. Creativity: Cognitive, personal, developmental, and social aspects. American Psychologist, 55(1):151-158, 2000.
- J. R. Suler. Primary process thinking and creativity. Psychological Bulletin, 88(1):144-165, 1980.
- G. A. Wiggins. A preliminary framework for description, analysis and comparison of creative systems. Knowledge-Based Systems, 19(7):449-458, 2006.
